# The euchromatic histone mark H3K36me3 preserves heterochromatin through sequestration of an acetyltransferase complex in fission yeast

**DOI:** 10.15698/mic2020.03.711

**Published:** 2020-01-03

**Authors:** Paula R. Georgescu, Matías Capella, Sabine Fischer-Burkart, Sigurd Braun

**Affiliations:** 1 Department of Physiological Chemistry, BioMedical Center (BMC), Ludwig-Maximilians-Universität München, Martinsried, Germany.

**Keywords:** chromatin, heterochromatin, silencing, acetyltransferase, histone modification

## Abstract

Maintaining the identity of chromatin states requires mechanisms that ensure their structural integrity through the concerted actions of histone modifiers, readers, and erasers. Histone H3K9me and H3K27me are hallmarks of repressed heterochromatin, whereas H3K4me and H3K36me are associated with actively transcribed euchromatin. Paradoxically, several studies have reported that loss of Set2, the methyltransferase responsible for H3K36me, causes de-repression of heterochromatin. Here we show that unconstrained activity of the acetyltransferase complex Mst2C, which antagonizes heterochromatin, is the main cause of the silencing defects observed in Set2-deficient cells. As previously shown, Mst2C is sequestered to actively transcribed chromatin via binding to H3K36me3 that is recognized by the PWWP domain protein Pdp3. We demonstrate that combining deletions of *set2*^*+*^ and *pdp3*^*+*^ results in an epistatic silencing phenotype. In contrast, deleting *mst2*^*+*^, or other members of Mst2C, fully restores silencing in Set2-deficient cells. Suppression of the silencing defect in *set2*Δ cells is specific for pericentromeres and subtelomeres, which are marked by H3K9me, but is not seen for loci that lack genuine heterochromatin. Mst2 is known to acetylate histone H3K14 redundantly with the HAT Gnc5. Further, it is involved in the acetylation of the non-histone substrate and E3 ubiquitin ligase Brl1, resulting in increased H2B-K119 ubiquitylation at euchromatin. However, we reveal that none of these mechanisms are responsible for the Set2-dependent silencing pathway, implying that Mst2 targets another, unknown substrate critical for heterochromatin silencing. Our findings demonstrate that maintenance of chromatin states requires spatial constraint of opposing chromatin activities.

## INTRODUCTION

The nucleus of eukaryotic cells is organized into topologically distinct chromatin domains, known as eu- and heterochromatin. Both are controlled through various post-translational histone modifications, nucleosome remodeling and RNA-related processes. Euchromatin is associated with active transcription and histone hyperacetylation, contributing to an open chromatin structure. In contrast, heterochromatin is associated with gene repression and histone hypoacetylation, often adopting a compact chromatin structure that restricts transcription and genomic recombination. Whereas constitutive heterochromatin is present at repeat-rich sequences like centromeres and telomeres, facultative heterochromatin can also form at gene-rich regions, e.g. during cellular differentiation and adaptation to environmental changes. Responding to these changes and maintaining the structural integrity of heterochromatin domains requires the concerted actions of histone modifiers, readers, and erasers [1–3].

A conserved type of heterochromatin is characterized by the presence of methylated lysine 9 of histone H3 (H3K9me) [2]. In *Schizosaccharomyces pombe*, distinct heterochromatin domains are present at pericentromeres, subtelomeres and the silent mating type locus. H3K9me is deposited by the sole histone methyltransferase Clr4 that is present in a complex known as CLRC (Clr4 methyltransferase complex) and catalyzes all three steps of methylation [4–6]. The repressive H3K9me mark is recognized by chromodomain-containing proteins, such as Clr4 itself [7] and members of the HP1 family that interact with various chromatin factors [8, 9]. Among those is the repressor complex SHREC (Snf2/Hdac repressive complex) that deacetylates histone H3 at lysine K14 (H3K14ac) and restricts access of RNA polymerase II (RNAPII) to heterochromatin [10–12].

Heterochromatin assembly is guided by several targeting mechanisms among which the RNA interference machinery plays a prominent role in *S. pombe* [13, 14]. Conversely, euchromatin is protected from ectopic heterochromatin assembly by several heterochromatin-antagonizing factors. The JmjC protein Epe1 counteracts H3K9me formation at euchromatic sites prone to heterochromatin assembly [15–17] and prevents spreading beyond heterochromatin boundaries [18]. Epe1 is recruited to HP1 proteins and competes with SHREC for HP1 binding, thereby facilitating access of RNAPII to chromatin [19–21]. Heterochromatin is further antagonized by the RNA polymerase RNAPII-associated factor 1 complex (Paf1C), which is involved in multiple steps in transcription. Mutants of Paf1C are susceptible to small interfering RNA (siRNA)-mediated heterochromatin initiation at ectopic sites, possibly due to altered kinetics in the processing and termination of nascent transcripts [22–24]. Paf1C also affects heterochromatin maintenance through its subunit Leo1, which prevents spreading at heterochromatin boundaries and promotes histone turnover [25, 26]. Furthermore, Paf1C's elongation function may help to overcome the repressive activity of H3K9me3 by supporting RNAPII in disrupting nucleosomes [27].

Histone acetyltransferases (HATs) also counteract heterochromatin by altering the charge and structure of nucleosomes, and also through the recruitment of factors to acetylated histones. The lysine acetytltransferase (KAT) Mst2 mediates H3K14 acetylation redundantly with the HAT Gcn5, which is part of the SAGA (Spt-Ada-Gcn5 acetyltransferase) complex [28]. Loss of Mst2 enhances silencing at subtelomeres [29] and bypasses the need for RNA interference (RNAi) in centromeric heterochromatin maintenance [30]. Furthermore, the rate at which ectopic silencing is initiated in a *paf1* mutant is drastically increased when Mst2 is absent [31]. Mst2 is present in a complex (Mst2C) homologous to *Saccharomyces cerevisiae* NuA3b, which contains the PWWP domain protein Pdp3 [28, 32]. Pdp3 binds to trimethylated H3K36 (H3K36me3) and sequesters Mst2 to actively transcribed chromatin [31, 32]. Notably, in Pdp3-deficient cells, Mst2 gains promiscuous access to heterochromatin, where it triggers a silencing defect [31]. However, none of these heterochromatin-associated phenotypes are recapitulated by the loss of Gcn5, implying that Mst2 has another, non-redundant function that involves an acetylation substrate other than H3K14 [30, 31]. Proteome analysis revealed that Mst2 is involved in the acetylation of Brl1, which is part of the histone ubiquitin E3 ligase complex (HULC). However, whether Brl1 acetylation is also responsible for the silencing defect under conditions when Mst2 encroaches on heterochromatin (i.e. in *pdp3*Δ cells) remains unknown.

H3K36 methylation is associated with actively transcribed chromatin. In budding and fission yeast, all three methylation states are mediated by a single enzyme, Set2 [33]. Set2 binds to the phosphorylated C-terminal domain (CTD) of transcribing RNAPII through its Set2 Rpb1 interacting (SRI) domain, which is a prerequisite for H3K36 tri- but not dimethylation [34, 35]. While H3K36 methylation is coupled to transcriptional elongation, it is also implicated in gene repression. In budding yeast, the histone deacetylase (HDAC) complex Rpd3S promotes histone deacetylation in the wake of transcribing RNAPII, which prevents initiation of aberrant transcription from cryptic promoters within coding regions. Rdp3S is recruited to chromatin through its interaction with the phosphorylated CTD of RNAPII [36]. In addition, the chromodomain subunits Eaf3 and Rco1 recognize di- and trimethylated H3K36 [37–40], which stimulates the HDAC activity of Rpd3S [36]. The fission yeast homologs of Rpd3S and Eaf3 are Clr6 complex II (Clr6C-II) and Alp13, respectively [41]. Mutants deficient in Set2 and Alp13 display increased antisense transcription in coding regions [41] and silencing defects at various heterochromatin domains [34, 42, 43]. Set2 is further required for the repression of subtelomeric regions, characterized by highly condensed chromatin bodies termed ‘knobs', which lack H3K9me and most other histone modifications [44]. However, whether silencing defects in *set2*Δ cells are mediated directly through a local loss of H3K36me or an alternative mechanism remains unclear.

Here we demonstrate that the silencing defects in *set2*Δ cells at canonical heterochromatin can be fully reversed by concomitant deletion of *mst2*^+^. Full suppression of the silencing defect is also seen in the absence of other Mst2C members, which are critical for proper complex assembly, but not for Pdp3 that recruits Mst2C to actively transcribed regions via H3K36me3. Together, our findings implicate that the silencing defect of *set2*Δ cells is caused by the global mislocalization of Mst2C and its encroachment on heterochromatic regions.

## RESULTS

### Deletion of the *mst2*^+^ gene suppresses the silencing defect of Set2-decifienct cells

**Figure 1:**
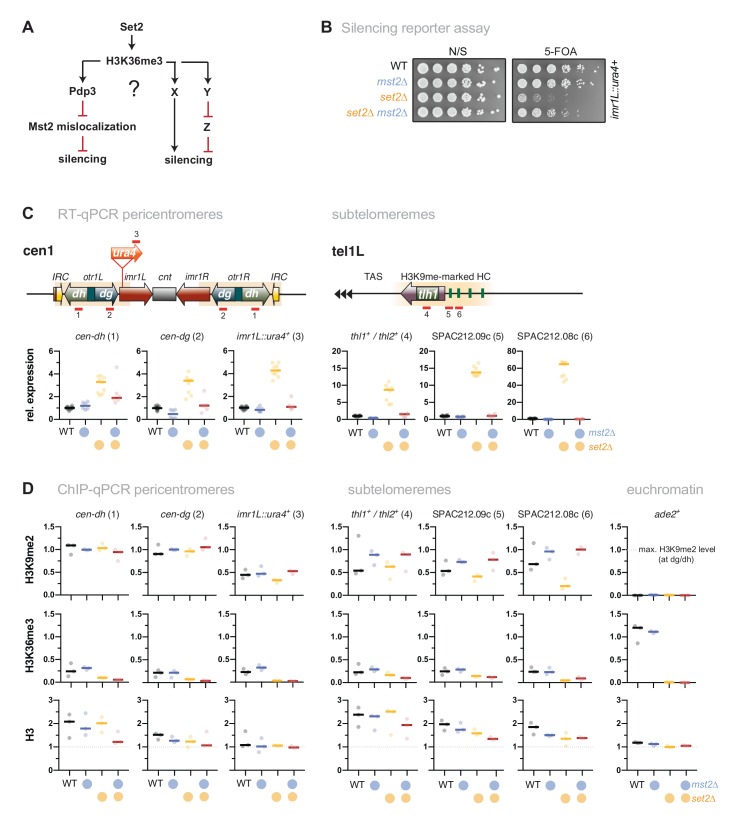
FIGURE 1: Loss of Mst2 recues the silencing defect caused by *set2*^*+*^ deletion. **(A)** Scheme depicting genetic interactions of *set2*^*+*^, *pdp3*^*+*^ and *mst2*^*+*^ contributing to heterochromatic silencing and potential parallel pathways in which H3K36me3 may be also involved. Black lines indicate positive regulations, red lines indicate negative regulations. **(B)** Silencing reporter assay with the *imr::ura4*^*+*^ reporter. Fivefold serial dilutions of wild-type (WT) cells and single and double deletion mutants of *mst2*^*+*^ and *set2*^*+*^; (N/S) nonselective medium. **(C)** RT-qPCR analysis. Shown are heterochromatic transcript levels of the strains used in (B). The schemes display the positions of the *ura4*^*+*^ reporter insertion and endogenous heterochromatic transcripts from pericentromeric (left) and subtelomeric heterochromatin (right); transcript levels have been normalized to *act1*^*+*^ and are shown relative to WT for each transcript. Circles and horizontal lines represent individual data and median from 6-12 independent experiments. **(D)** ChIP-qPCR analysis for H3K9me2 (top), H3K36me3 (middle) and H3 (bottom) at pericentromeric and subtelomeric heterochromatin; *ade2*^*+*^ (right panels) was used as control for euchromatin. Circles and horizontal lines represent individual data and median from 3 independent experiments. Input-normalized ChIP data were corrected for variation in IP efficiency by normalizing to the mean of *cendg* and *cendh* for H3K9me2, or the mean of three euchromatic loci (*tef3*^*+*^, *ade2*^*+*^, *act1*^*+*^) for H3K36me3 and H3. Note that H3K9me2 is largely unaltered at the *dh* repeats in *set2*Δ [34].

We previously showed that loss of the PWWP subunit Pdp3 causes moderate silencing defects of the pericentromeric *imr1L::ura4*^*+*^ reporter gene and various subtelomeric genes, which are suppressed when *mst2*^+^ is concomitantly deleted [31]. Since Pdp3 anchors Mst2 to euchromatin via H3K36me3 [31], we tested whether the silencing defects of *set2*Δ at various pericentromeric and subtelomeric heterochromatic loci [34, 41–44] can be attributed to Mst2C mislocalization. This hypothesis makes the prediction that silencing will be restored when Mst2 is eliminated in *set2*Δ cells, analogous to *mst2*^+^ deletion in a *pdp3*Δ strain (see scheme, **Figure 1A**). Silencing can be monitored *in vivo* using the *ura4*^*+*^ reporter gene inserted into a heterochromatic region. Presence of the nucleotide analog 5-FOA (5′-fluoroorotic acid) inhibits cell growth due to the conversion of 5-FOA into a toxic metabolite by the gene product of *ura4*^*+*^ but allows growth when *ura4*^*+*^ transcription is repressed. By examining pericentromeric silencing in the *imr1L::ura4*^*+*^ reporter strain used previously [31], we found that growth of *set2*Δ cells on 5-FOA is impaired, similar to *pdp3*Δ cells and consistent with other studies that have reported silencing defects for *set2*Δ [34, 41–43]. Remarkably, while cell growth in the presence of 5-FOA was not affected by lack of Mst2, it was nearly restored when *mst2*^*+*^ was deleted in a *set2*Δ background (**Figure 1B**). We confirmed the findings of the reporter assay by reverse transcriptase assays combined with quantitative PCR (RT-qPCR). Deletion of *set2*^*+*^ causes a reproducible upregulation of the *imr1L::ura4*^*+*^ reporter gene (4-fold) and two endogenous transcripts from the outer *dg* and *dh* repeats (both 3-fold; **Figure 1C**, left panels). In contrast, transcript levels in the *set2*Δ *mst2*Δ double mutant resemble those of wild-type (WT) cells. In addition, we examined expression levels of transcripts derived from a subtelomeric region that is marked with high levels of H3K9me2 (10-50 kb distal of the telomeric repeats, known as subtelomeric heterochromatin). De-repression of the subtelomeric genes *tlh1*^*+*^/*tlh2*^*+*^, *SPAC212.09c* and *SPAC212.08c* in *set2*Δ cells is even more pronounced (10-, 15- and 60-fold, respectively; **Figure 1C**, right panels) than what we found at pericentromeres. Nonetheless, transcriptional upregulation at these loci is completely suppressed when *mst2*^*+*^ is concomitantly deleted in *set2*Δ cells.

Constitutive heterochromatin in *S. pombe* is marked by high levels of H3K9me but largely devoid of euchromatic histone modifications (i.e. H3K4me, H3K36me) [43, 45]. By performing chromatin immunoprecipitation coupled to quantitative PCR (ChIP-qPCR), we observed a moderate H3K9me2 decrease in *set2*Δ cells for several heterochromatic loci that display intermediate H3K9me2 levels (*imr1L::ura4*^*+*^ at pericentromeres; *SPAC212.09c* and *SPAC212.08c* at subtelomeres; **Figure 1D**, upper panels). Conversely, *mst2*Δ and *set2*Δ *mst2*Δ cells showed elevated H3K9me2 levels at those loci, suggesting that Mst2 counteracts H3K9me2 in a chromatin context-dependent manner. This notion was further supported by a comprehensive analysis of H3K9me2 using tiling oligonucleotides covering pericentromeric and subtelomeric heterochromatin, and a facultative heterochromatin island at the *mei4*^*+*^ locus (Figure S1). Especially at telomere-distal heterochromatin (30-50 kb downstream of telomeric repeats) and *mei4*^*+*^, loss of Mst2 resulted in a large H3K9me2 increase, suggesting that those chromatin regions are particularly prone to heterochromatin assembly.

While H3K4me is completely absent at constitutive heterochromatin [45], residual levels of H3K36me3 have been detected, particularly in the S phase during which pericentromeric repeats are preferentially transcribed [34, 43]. We also found low H3K36me3 levels at pericentromeres and subtelomeres, reaching only 10-20% of the enrichment observed at euchromatin (**Figure 1D**, middle panels; note that the absolute level might even be lower, since the anti-H3K36me3 antibody used here shows limited cross-reactivity with H3K9me2; Figure S2). Importantly, H3K36me3 levels changed only marginally in *set2*Δ and *mst2*Δ single mutants at most heterochromatin loci examined and, as expected, remained low in cells lacking both Set2 and Mst2, despite of silencing being restored in the double mutant. We also analyzed nucleosome abundance by examining (total) histone H3. While H3 ChIP enrichments tend to be lower for some loci in these mutants, most changes were not significant and did not reflect transcriptional upregulation or changes in histone modifications (**Figure 1D**, lower panels). Together, these findings suggest that the silencing defects at pericentromeric and subtelomeric heterochromatin are not primarily caused by local changes of H3K36me3 within heterochromatin (which is already low in WT cells). Rather, our results imply that they are triggered by the uncontrolled activity of Mst2, which in the absence of Set2 is no longer tethered to euchromatin and gains promiscuous genome-wide access to chromatin, including the heterochromatic regions [31].

### Set2 acts in the same genetic pathway as other Mst2C members

**Figure 2:**
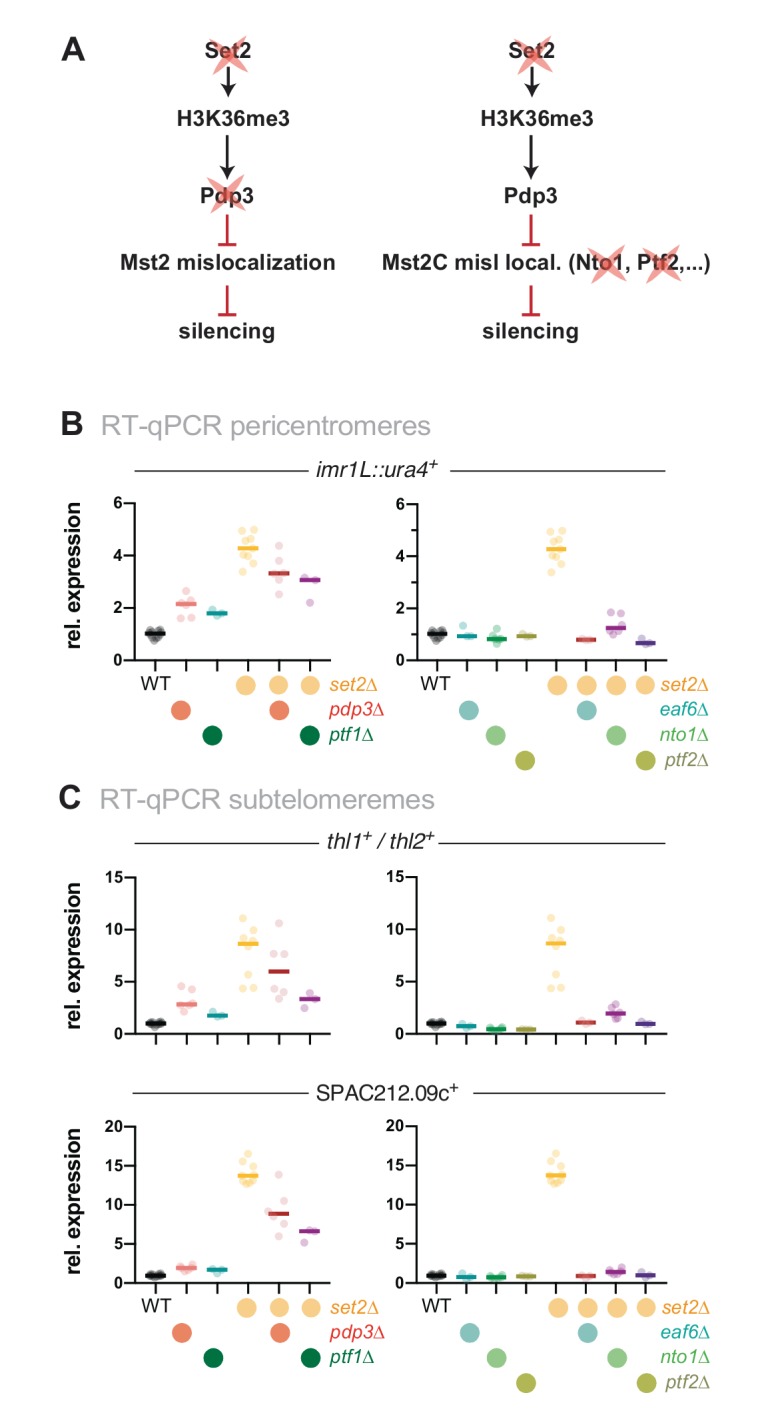
FIGURE 2: Loss of heterochromatin silencing in *set2*Δ is dependent on functional Mst2C. **(A)** Scheme displaying genetic interactions and genes mutated for experiments shown in (B) and (C). **(B, C)** RT-qPCR analysis of transcript levels at pericentromeric (B) and subtelomeric HC (C) RT-qPCR data analysis and primer positions as in Figure 1C. Circles and horizontal lines represent individual data and median from 6 independent experiments unless specified (i.e. WT: *n* = 12; *ptf1Δ, ptf2Δ, eaf6Δ, ptf1Δ set2Δ, ptf2Δ set2Δ,* and *eaf6Δ set2Δ*: *n* = 3).

We previously demonstrated that the Mst2C subunit Pdp3 mediates Mst2 recruitment via its PWWP domain that binds to H3K36me3 (**Figure 2A**, left panel) [31]. Since Set2 acts upstream of Pdp3 in Mst2 recruitment, we tested whether Set2 and Pdp3 also participate in the same pathway with respect to heterochromatin silencing. In agreement with our former findings [31], cells lacking Pdp3 displayed alleviated silencing at pericentromeres and subtelomeres, although we noted that the transcriptional increase was less pronounced in *pdp3*Δ than in *set2*Δ (**Figure 2B** and **C**, left panels). Combining both deficiencies did not result in an additive increase; we rather observed a mild suppressive phenotype for *set2*Δ *pdp3*Δ when compared to the *set2*Δ single mutant. Although the nature of the partial suppression remains unclear (see discussion), the non-additive phenotype of the double mutant suggests that Pdp3 and Set2 act in the same pathway.

Besides Mst2 and Pdp3, Mst2C contains five additional subunits: Nto1, Eaf6, Tfg3, Ptf1 and Ptf2. Eaf6 is also present in the NuA4 acetyltransferase complex, while Tfg3 is shared with Ino80, SWI/SNF and the TFIID and TFIIF complexes. The functions of these subunits within Mst2C are not well understood, but Nto1 and Ptf2 are essential for the integrity and assembly of the complex, and mutants lacking either of these subunits phenocopy the loss of Mst2 [28]. We therefore tested whether deleting those subunits, analogous to *mst2*^*+*^ elimination, suppresses the silencing defect of *set2*Δ (**Figure 2A**, right panel). Similar to *mst2*Δ, eliminating *nto1*^*+*^, eaf3^+^ and *ptf2*^*+*^ alone did not increase gene repression but in combination with *set2*Δ completely suppressed the silencing defect (**Figure 2B** and **C**, right panels). In contrast, deletion of *ptf1*^*+*^ rather resembled the phenotype of *pdp3*Δ, showing a moderate upregulation and partial suppression in combination with *set2*Δ, suggesting that Ptf1 also contributes to the recruitment of Mst2C to H3K36me3 (**Figure 2B** and **C**, left panels). We refrained from examining the *tfg3*^*+*^, as its presence in multiple complexes likely causes pleiotropic effects that may be difficult to interpret. From these data we conclude that an intact Mst2 complex is required to trigger the silencing defect at heterochromatin.

### Mst2-dependent silencing defects are not mediated through Brl1 acetylation

**Figure 3:**
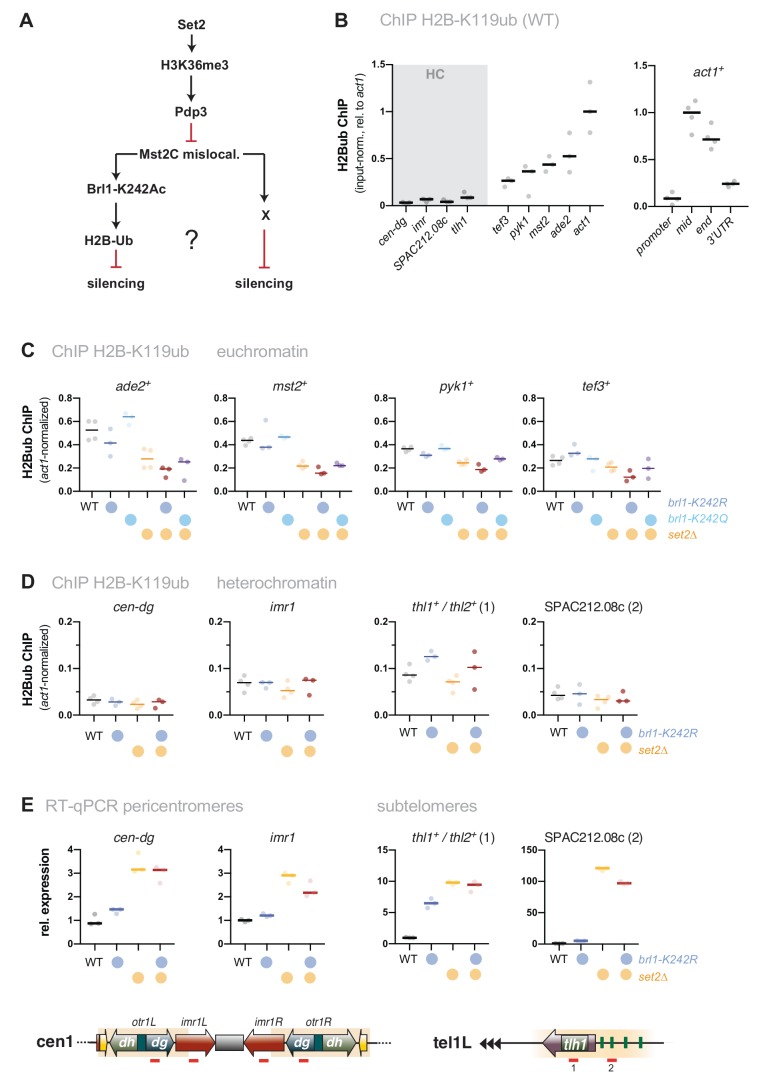
FIGURE 3: The target of Mst2 in heterochromatin is not Brl1. **(A)** Scheme depicting the described Mst2C pathway (Flury et al., 2017) involving Brl1-K242 acetylation and H2B ubiquitylation and a potential alternative pathway on HC silencing; black arrows represent positive regulation and red lines represent negative regulation. **(B)** ChIP-qPCR analysis for H2B-K119ub in wild-type cells. Circles and horizontal lines represent individual data and median from 3 (left panel) and 4 independent experiments (right panel). Input-normalized ChIP data are shown relative to the median of the ChIP signals for *act1*^*+*^ (mid). (**C, D**) ChIP-qPCR analysis for H2B-K119ub in mutants affecting Brl1 acetylation. Circles and horizontal lines represent individual data and median from 3 independent experiments (except: WT, *set2Δ*: *n* = 4). ChIP analysis as in (B), except that ChIP data were corrected for variation in IP efficiency by normalizing to *act1*^*+*^ (mid). Note that H2B-K119ub at *act1*^*+*^ is not largely affected by Set2. **(E)** RT-qPCR analysis of transcript levels at pericentromeric and subtelomeric HC. Data analysis as in Figure 1C (*n* = 3 individual experiments).

We previously showed that Mst2 is involved in the acetylation of the non-histone substrate Brl1, a conserved ubiquitin E3 ligase that mono-ubiquitylates histone H2B at lysine 119 [31]. Acetylation of Brl1 at lysine 242 (Brl1-K242ac) may have a stimulatory effect on its enzymatic activity (H2B-K119ub) and downstream events (H3K4me3), which protects euchromatic genes against the ectopic formation of heterochromatin, likely through increased transcription [31]. We therefore wondered whether the Brl1-K242ac-dependent positive feedback loop is the main cause for the silencing defect observed in *set2*Δ cells (**Figure 3A**). By ChIP, we confirmed previous reports [46, 47] that H2B-K119ub is low at heterochromatic regions but highly enriched over the gene body of actively transcribed genes (**Figure 3B**). Interestingly, H2B-K119ub was significantly reduced in the absence of Set2 at several of euchromatic genes but not at *act1*^*+*^ (Figure S3), which we used as an internal control for normalization in the subsequent ChIP experiments. We found that cells expressing the *brl1-K242R* mutant that inhibits Brl1 acetylation also showed decreased H2B-K119ub at several euchromatic genes (*ade2*^*+*^, *mst2*^*+*^, *pyk1*^*+*^ but not *tef3*^*+*^, **Figure 3C**) yet less distinct than seen in *set2*Δ cells. The *set2*Δ *brl1-K242R* double mutant displayed a slightly enhanced decrease, whereas the *brl1-K242Q* mimicking Brl1 acetylation did neither alter H2B-K119ub in WT nor in *set2*Δ cells. Together, these data imply that mutations affecting Brl1 acetylation regulate H2B ubiquitylation in euchromatin, which is in agreement with our previous observation for total H2B-K119ub [31] (although it appears that Set2 also contributes through a Mst2-independent pathway).

However, when examining heterochromatin, we found hardly any change in these mutants for H2B-K119ub, which was 5-10 times lower than euchromatin in WT cells and remained low in *brl1-K242R* and *set2*Δ cells (**Figure 3D**). Moreover, in stark contrast to *set2*Δ *mst2*Δ cells (**Figure 1C**), we found that silencing at pericentromeres and subtelomeres was not reinstated in the *set2*Δ *brl1-K242R* double mutant (**Figure 3E**). Hence, preventing Brl1 acetylation is not sufficient to block the anti-silencing activity of Mst2. Instead, we observed transcriptional upregulation even in the single *brl1-K242R* mutant for some heterochromatic loci, suggesting that the loss of its acetylation target Brl1 (in euchromatin) renders Mst2 more active (in heterochromatin). From this we conclude that Mst2's euchromatin-protective role via Brl1-K242ac differs from its role in counteracting gene silencing at heterochromatin. This further implies that Mst2 targets at least one other substrate that is critical for heterochromatin silencing.

### Silencing defects in *set2*Δ at other repressed loci involve an Mst2-independent pathway

**Figure 4:**
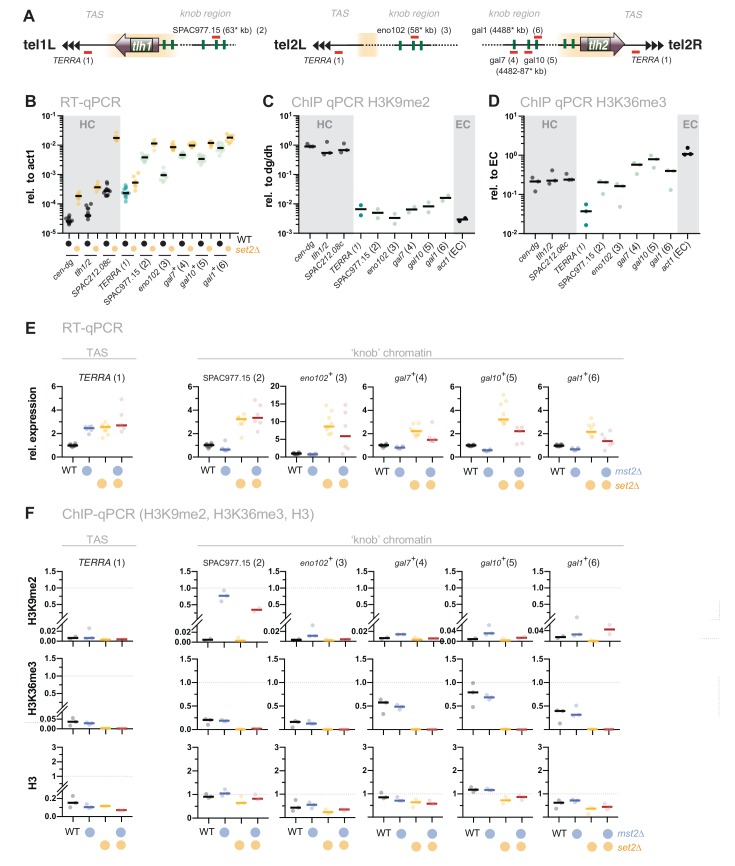
FIGURE 4: The silencing defect at ‘knobs' in *set2Δ* is not associated with the Mst2 pathway. **(A)** Scheme depicting expression sites of the non-coding RNA *TERRA* and the positions of several loci within the ‘knob' regions on chromosomes I and II. Chromosomal positions refer to annotations in www.pombase.org but differ from the absolute positions due to missing sequences at the chromosomal termini. **(B)** RT-qPCR analysis in WT and *set2Δ* strains comparing transcript levels at pericentromeric and subtelomeric HC (shaded in grey) to loci in the ‘knob' region. Transcript levels from 12 independent experiments are shown relative to *act1*^*+*^. (**C, D**) ChIP-qPCR analysis of H3K9me2 and H3K36me enrichments in WT cells at loci analyzed in (B). Shown are ChIP data from 2-3 individual experiments analyzed as described in Figure 1D. **(E)** RT-qPCR analysis in *mst2Δ* and *set2Δ* single and double mutants; data from independent experiments analyzed as described in Figure 1C (WT *n* = 12; *mst2Δ, mst2Δ set2Δ*: *n* = 6; *set2Δ*: *n* = 9). **(F)** ChIP-qPCR analysis of *TERRA* and different loci located within the ‘knob' region; data from independent experiments analyzed as described in Figure 1D (*n* = 3).

Loss of *set2*^*+*^ does not only affect gene repression at chromatin regions with high levels of H3K9me2 but also other subtelomeric loci. These include the telomere-proximal region (300 bp – 10 kb, known as telomere-associated sequences, TAS), as well as a region 50 kb downstream of the telomeric repeats, which is characterized by highly condensed chromatin bodies dubbed ‘knobs' [44] (**Figure 4A** and **B**). Transcription of the non-coding RNA *TERRA* (telomeric repeat-containing non-coding RNA) from the TAS is repressed by heterochromatin and members of shelterin, the telomere-end protecting complex [48, 49]. However, this subtelomeric region displays low nucleosome abundance [50] and establishes only low levels of H3K9me2 and H3K36me3 (**Figure 4C** and **D**). Similarly, subtelomeric ‘knob' genes are decorated with a low amount of H3K9me2 and display reduced H3K36me3 compared to euchromatin (**Figure 4C** and **D**).

When we examined gene expression at the TAS, we found a subtle but reproducible upregulation (2 to 3-fold) of *TERRA* in *set2*Δ. A similar increase was also observed in *mst2*Δ, and concomitant deletion of *mst2*^+^ in the *set2*Δ mutant did not suppress the silencing defect (**Figure 4E**, left panel). In agreement with previous studies, we also detected a 2 to 10-fold upregulation for several ‘knob' genes [34, 44]. Additional deletion of *mst2*^+^ did not restore silencing of these loci in *set2*Δ (or caused only a partial suppression; **Figure 4E**). Nonetheless, disruption of *mst2*^+^ promoted the establishment of H3K9me2 at several knob genes (particularly *SPAC977.15*), corroborating the notion of Mst2C playing a global role in antagonizing heterochromatin [30, 31, 51]. However, since removal of Mst2 was not sufficient to reinstate silencing in the absence of Set2, we presume that an additional, Mst2- and H3K9me-independent pathway interferes with the repression of these genes.

## DISCUSSION

### Set2-dependent silencing defects in heterochromatin are functionally linked to Mst2C

Transcriptionally active and repressed chromatin regions are marked by different posttranslational modifications. H3K36me3 in particular is deposited co-transcriptionally through the recruitment of Set2 by transcribing RNAPII and likely other elongating factors [35]. Yet paradoxically, loss of Set2 also causes defects in transcriptionally repressed heterochromatin [34, 41–43]. We previously have shown that deleting *mst2* restores silencing in cells lacking the H3K36me3 reader Pdp3 [31]. Here, we demonstrate that eliminating the catalytic KAT subunit Mst2, or other subunits critical for Mst2C assembly, fully reverses the silencing defect of Set2-deficient cells at constitutive heterochromatin regions (**Figure 1** and **2**). Reinstatement of silencing is not only seen at the level of transcription but also at the level of heterochromatin structure (H3K9me2) at loci where loss of Set2 affects both (**Figure 1**). In contrast, H3K36me3 is low at these heterochromatin regions even in WT cells and also not affected upon deletion of *mst2*^+^. This suggests that defective silencing is not caused directly by the loss of H3K36me within heterochromatin but rather indirectly through the promiscuous activity of Mst2C. Although Set2 is implicated in various cellular functions, pleiotropic defects appear to be less likely the cause of impaired silencing. Instead, the fact that silencing is completely restored at constitutive heterochromatin implies that Set2 exclusively controls gene repression through sequestration of Mst2 by H3K36me3. It is worth mentioning that although Mst2 is not detected by ChIP at transcribed chromatin in *pdp3*Δ cells, it has still access to chromatin, as we previously demonstrated by DamID experiments [31].

Recruitment of Mst2 to euchromatin is mediated by the Mst2C subunit Pdp3, which binds to H3K36me3 via its PWWP domain. Consistently, lack of Pdp3, or a point mutation within its PWWP domain, also produces a defect in heterochromatin silencing [31]. However, we noticed that the silencing defect in *pdp3*Δ is less pronounced than in *set2*Δ (**Figure 2**), despite the fact that silencing can be fully restored in the absence of Mst2 (see **Figure 1**). A possible explanation would be that Mst2 recruitment involves another H3K36me3-binding factor that acts redundantly with Pdp3. Indeed, the Mst2C subunit Nto1 contains two PHD (plant homeodomain) fingers, and the *S. cerevisiae* homolog shows affinity for H3K36me3 [52]. However, since Nto1 is essential for Mst2C assembly, a putative role in restricting Mst2 to euchromatin would be masked by the complete loss of KAT activity, resulting in deviating phenotypes for *pdp3*Δ and *nto1*Δ mutants. Alternatively, Pdp3 may also contribute to the stability or activity of the complex (at least in part), in addition to its function in H3K36me3 anchoring. This could explain the intermediate phenotype of *pdp3*Δ cells compared to *set2*Δ on one hand, and *mst2*Δ on the other. More work will be needed to better understand the functions of the individual subunits of Mst2C.

### Mst2C has distinct cellular functions by acetylating multiple targets

Mst2 acetylates H3K14 *in vitro* and *in vivo* and acts redundantly with the SAGA member Gcn5 [28]. H3K14ac is critical for G2/M checkpoint activation upon DNA damage and controls chromatin compaction through recruitment of the nucleosome remodeler RSC [28]. H3K14ac also accumulates in heterochromatin upon deletion of the HDAC Clr3 and other components of the repressor complex SHREC, suggesting a function in antagonizing heterochromatin silencing [10, 53]. Moreover, the anti-silencing factor Epe1 physically interacts with SAGA and targets the HAT to heterochromatin when Epe1 is overexpressed. This triggers a silencing defect that is accompanied by an H3K14ac increase and is dependent on the HAT activity of Gcn5 [54]. At first glance, this seems reminiscent of the phenotype caused by relocalization of Mst2. However, additional findings cast doubt on whether H3K14 is the relevant substrate that mediates the anti-silencing function of Mst2. First, neither lack of Set2 nor of Pdp3 (both causing delocalization of Mst2) results in H3K14ac accumulation at heterochromatin, as we and other showed previously [31, 34]. Second, while elimination of Mst2 bypasses the need for RNAi in pericentromeric silencing, this is not the case for mutants lacking Gcn5 [30]. Third, lack of Mst2 but not Gcn5 promotes the assembly of ectopic heterochromatin domains [31]. Together, these findings suggest a non-redundant function of Mst2 in antagonizing heterochromatin that likely involves another substrate than H3K14.

**Figure 5:**
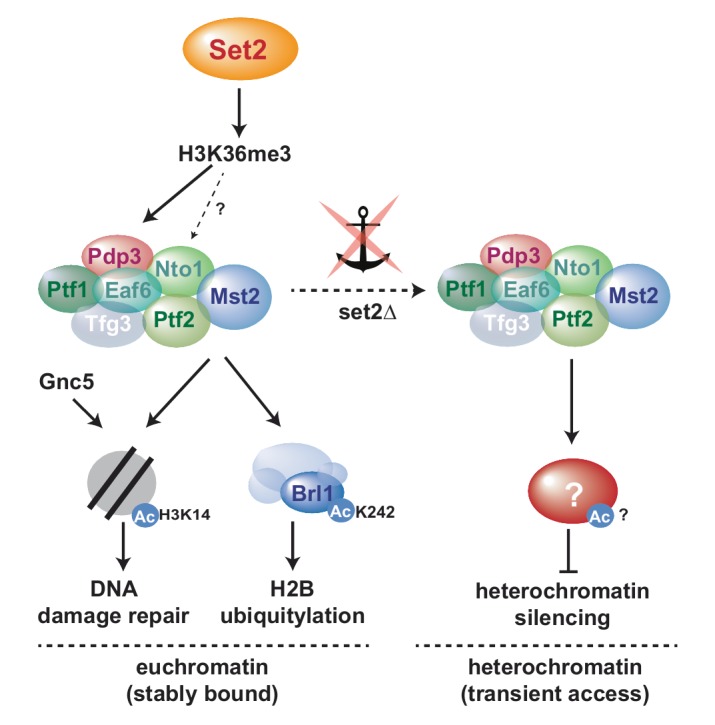
FIGURE 5: Model for Mst2C-dependent functional pathways in the presence of H3K36me3 (sequestered to euchromatin) and in the absence of H3K36me3-mediated anchoring (promiscuous access to heterochromatin).

We previously showed that the euchromatin-protective role of Mst2 is mediated through acetylation of the non-histone substrate Brl1, a subunit of HULC [31]. In particular, replacing *brl1*^*+*^ with an acetylation-deficient mutant (*brl1-K242R*) phenocopied the deletion of *mst2*^*+*^, whereas mimicking acetylation (*brl1-K242Q*) bypassed the need for Mst2 in protecting euchromatin. In agreement with this finding, deletion mutants of *brl1*^*+*^ or other components of HULC display more robust heterochromatin silencing than WT cells [47]. Thus, acetylated Brl1 seemed to be a likely candidate for mediating the anti-silencing function of Mst2 also at heterochromatin. However, in contrast to *mst2*Δ, we found that introducing the *brl1-K424R* mutant was not sufficient to suppress the silencing defect of *set2*Δ. While initially we did not expect a Brl1-independent Mst2 function at heterochromatin, several lines of evidence support the idea that Mst2 acts through different pathways at eu- and heterochromatin. First, whereas Mst2 antagonizes heterochromatin even when not recruited by H3K36me3, Brl1 acetylation and euchromatin protection requires stable chromatin binding of Mst2 via Pdp3 [31]. Consistently, we found that H2B-K119ub levels are altered in set2 and brl1 mutants at several euchromatic genes but not at heterochromatin (**Figure 3C** and **D**). Second, at heterochromatin, loss of Pdp3 or Set2 causes de-repression and H3K9me decrease, whereas lack of Mst2 results in enhanced repression and increased H3K9me. However, at euchromatin and facultative heterochromatin, the lack of either Pdp3 or Mst2 causes ectopic heterochromatin assembly (Figure S1; [31]). Finally, for several subtelomeric genes, we observed a synthetic defect in gene repression when combining *set2*Δ with the acetylation-mimicking *brl1-K242Q* mutant, suggesting that both pathways can affect these loci (Figure S4). We therefore speculate that besides H3K14 and Brl1-K242 Mst2C targets at least one other substrate that is important for heterochromatin maintenance (**Figure 5**). Previous proteomics failed so far to identify additional substrates besides Brl1 that involve acetylation by Mst2 [31]. However, since heterochromatin makes up only a small portion of the genome and Mst2 may have only transient access to these genomic regions, a heterochromatin-specific substrate would be more difficult to identify.

### Set2-mediated gene repression at chromatin lacking H3K9me is independent of Mst2C

Besides defects at constitutive heterochromatin, deletion of *set2*^*+*^ results in the transcriptional upregulation of other genes that are part of repressed chromatin regions but largely devoid of H3K9me2. These include the non-coding RNA *TERRA* expressed from the telomere-proximal TAS region as well as various other genes expressed from the telomere-distal ‘knob' region, which is characterized by high chromatin condensation and the absence of most post-transcriptional histone modifications [44]. However, except for *TERRA*, we still detect some H3K36me3 at these chromatin regions, albeit reduced compared to euchromatin (**Figure 4**). Transcriptional upregulation of these loci is moderate in *set2*Δ and not suppressed by concomitant Mst2 elimination. Nonetheless, Mst2 may still gain access to these loci and antagonize heterochromatin formation, as seen by increased levels of H3K9me2 upon *mst2*^+^ deletion. Nonetheless, the lack of suppression suggests the involvement of a Set2-dependent but Mst2-independent pathway that is critical for gene repression.

Another histone-modifying complex potentially involved in Set2-dependent silencing is the HDAC Clr6C-II, which contains the chromodomain protein Alp13 (Eaf3 in *S. cerevisiae*; MORF4 in humans) [41, 55]. The homologous complex in *S. cerevisiae*, Rpd3S, prevents cryptic transcription through deacetylation of histones in coding regions marked with H3K36me2/3 via Eaf3 [36–39]. Similarly, Clr6C-II promotes deacetylation of H3K9 in coding regions, and both Alp13 and Set2 prevent antisense transcription and repress pericentromeric repeats [41]. In addition, both H3K36me3 and Alp13 accumulate on heterochromatin during S phase when pericentromeric repeats are transcribed [43]. However, in contrast to its *S. cerevisiae* homolog, *S. pombe* Set2 does not contribute to deacetylation of bulk histones and only moderately affects antisense transcription; in addition, the *set2*Δ *alp13*Δ double mutant displays an additive defect, thus arguing for parallel pathways [41]. Moreover, while H3K36me2 is sufficient to recruit budding yeast Rpd3S [56], heterochromatin silencing and recruitment of Pdp3/Mst2C requires H3K36me3 [31, 34]. Together with the fact that silencing is fully restored in the absence of Mst2, it therefore appears less likely that Clr6C-II contributes significantly to the Set2-dependent pathway at heterochromatin. Still, it remains an attractive hypothesis that Clr6C-II represses transcription in an H3K36me/Alp13-dependent manner at other loci where Mst2 plays a less prevailing role.

### H3K36me-mediated KAT sequestration is conserved in heterochromatin maintenance

In worm embryos, perinuclear heterochromatin is established through two methyltransferases (MET-2, SET-25) and the nuclear membrane-associated chromodomain protein CEC-4, which tethers H3K9me-marked chromatin to the nuclear periphery [57, 58]. However, during differentiation, this pathway becomes redundant. An RNAi screen in *cec-4*-null larvae identified the chromodomain protein MRG-1 as a critical factor for perinuclear heterochromatin organization [59]. MRG-1 is homologous to the Rpd3S subunit Eaf3 (*S. cerevisiae*) and Clr6C-II subunit Alp13 (*S. pombe*). In addition, MRG-1 may associate with the HAT CBP-1/p300 analogous to other organisms. Although MRG-1/CBP-1 and Pdp3/Mst2C are not homologous to each other, there are still striking parallels [59]: Like Pdp3, MRG-1 binds to H3K36me3-marked euchromatin. Loss of perinuclear heterochromatin in *mrg-1*-null is phenocopied by the double mutant lacking the H3K36 methyltransferases MET-1 and MES-4. Reducing CBP-1 restores silencing in *mrg-1*-null larvae, whereas overexpression of *cbp-1* is sufficient to release heterochromatin from the nuclear periphery in the absence of CEC-4. Moreover, the authors detected increased CBP-1 binding at several heterochromatic genes in *mrg-1*-null larvae. This was accompanied by elevated histone acetylation (H3K27ac), providing a direct link to gene expression.

Altogether, this demonstrates that the principle of heterochromatin maintenance through internal sequestration of KATs is conserved between fission yeast and worms, despite some apparent differences regarding the molecular mechanisms (i.e. the nature of the KAT enzymes and their substrates). It further unveils that the pathways partition in eu- and heterochromatin are remarkably entwined, requiring spatial constraint of opposing chromatin activities to maintain the identity of chromatin states. Recent observations have reinforced the notion that repressive histone marks contribute to epigenetic inheritance of chromatin domains [15, 16, 27, 60]. In contrast, histone modifications associated with euchromatin have been considered rather a consequence than a cause of transcription. The discovery of H3K36me3 as a critical factor in heterochromatin maintenance will likely reopen the discussion to what extent ‘active’ marks also contribute to the epigenetic states of chromatin.

## MATERIALS AND METHODS

### Contact for reagent and resource sharing

Important reagents and assays used are listed in Table S5. Further information and requests for resources and reagents should be directed to and will be fulfilled by Sigurd Braun (sigurd.braun@bmc.med.lmu.de).

### Yeast techniques and strains

Standard media and genome engineering methods were used [61]. For the *ura4*^*+*^ reporter assay in **Figure 1B** cells were plated on EMM or EMM containing 1 mg/mL FOA. The strains were grown at 30°C for three (non-selective, NS) and four days (5-FOA), respectively. Cultures were grown at 30°C in liquid YES media (160 rpm, 12-24 hours) or at 30°C on solid YES agarose plates (for 3 days). The *brl1-K242R* point mutant was provided by M. Bühler (FMI, Basel). Strains used in this study are listed in Table S1.

### RT-qPCR analyses

RT-qPCR experiments were carried out as previously described [62]. The data are presented as individual data points together with the median. cDNA was quantified by qPCR using the primaQUANT CYBR Master Mix (Steinbrenner Laborsysteme) and a QuantStudio 5 Real-Time PCR System (Applied Biosystems) and primers listed in Table S2. Prior to calculation of the median, *act1*^*+*^ normalized data sets from independent experiments were standardized to the mean of all samples from each experiment (experimental normalization; *eq 1.1*). These sample pool-normalized results were shown relative to the mean value of the sample pool-normalized WT data from all (n) experiments (*eq 1.2*).

Experimental normalization:

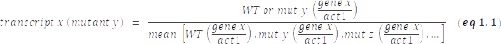


Mean WT normalization:

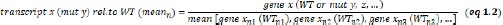


Using the average from a collection (sample pool) instead of a single strain (e.g. WT) reduces bias, especially when transcripts levels are low in the repressed state and therefore more prone to noise.

### ChIP assays

ChIP experiments were performed essentially as described [21]. Cross-linking was performed with 1% formaldehyde for 10 min at room temperature. For quantitative ChIP, immunoprecipitations were performed with 2 µ#x03BC;g of the following antibodies (cell lysates corresponding to different amounts of OD_600_): anti-H3K9me2 (15 OD_600_) anti-H3K36me3 (5 OD_600_), anti-H3 (5 OD_600_) and anti-H2B-K120ub (20 OD_600_). Antibodies are listed in Table S4. Immunoprecipitated DNA was quantified by qPCR using the primaQUANT CYBR Master Mix (Steinbrenner Laborsysteme) and a QuantStudio 5 Real-Time PCR System (Applied Biosystems). Primers are listed in Table S2. Unless otherwise noted, the median was calculated from three independent experiments. qPCR signals were normalized against the input samples for each primer position as internal control. For ChIP experiments with anti-H3K9me2, the input-normalized values were corrected for variation by normalizing against the mean of *cen-dg* and *cen-dh* as the *otr* is the region with the highest and most stable H3K9me2 enrichment (‘HC normalized', *eq 2.1*). For ChIP experiments with anti-H3K36me3 and H3, input-normalized qPCR signals were normalized to the mean of 3 euchromatic loci (*act1*^*+*^, *ade2*^*+*^, *tef3*^*+*^) as an internal control, which was set to 1 (‘EC normalized', *eq 2.2*). ChIP results with anti-H2B-K119ub were analyzed analogously, except that ChIP signals were normalized to input (**Figure 3B** and Figure S3) or *act1*^*+*^ as an internal control (**Figure 3C** and **D**).

Internal ChIP normalization H3K9me2:

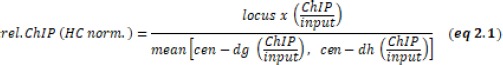


Internal ChIP normalization H3K6me3 and H3:

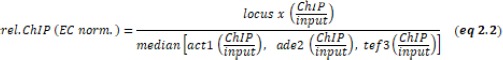


Using the mean of multiple euchromatic loci (‘EC') instead of single locus (e.g. *act1*^*+*^) reduces bias coming from variations in ChIP experiments, especially when doing IP experiments with bulk histones.

## AUTHOR CONTRIBUTIONS

PRG and SB designed the study. PRG generated yeast strains and performed RT-qPCR experiments with assistance by SFB. PRG and MC performed ChIP-qPCR experiments with assistance by SFB. MC performed silencing reporter assays. PRG and SB analyzed all data. SB wrote the manuscript, and PRG and MC contributed to editing.

## SUPPLEMENTAL MATERIAL

Click here for supplemental data file.

All supplemental data for this article are available online at http://www.microbialcell.com/researcharticles/2020a-georgescu-microbial-cell/.

## Abbreviations:

5-FOA: – 5′-fluoroorotic acid,
ChIP: – chromatin immunoprecipitation,
CTD: – C-terminal domain,
HAT: – histone acetyltransferase,
HDAC: – histone deacetylase,
HULC: – histone ubiquitin E3 ligase complex,
KAT: – lysine acetytltransferase,
Mst2C: – Mst2 complex,
qPCR: – quantitative PCR,
RNAi: – RNA interference,
RNAP: – RNA polymerase,
SAGA: – Spt-Ada-Gcn5 acetyltransferase,
SHREC: – Snf2/Hdac repressive complex,
TAS: – telomere-associated sequence,
TERRA: – telomeric repeat-containing non-coding RNA,
WT: – wild-type.
