# Supplementary material for: The euchromatic histone mark H3K36me3 preserves heterochromatin through sequestration of an acetyltransferase complex in fission yeast
Source: Microb Cell. 2020 Jan 3;7(3):80–92. doi: 10.15698/mic2020.03.711 (PMC7052950; doi:10.15698/mic2020.03.711)
Supplement: Supplementary file 1 [file mic-07-080-s01.pdf]

Figure S1

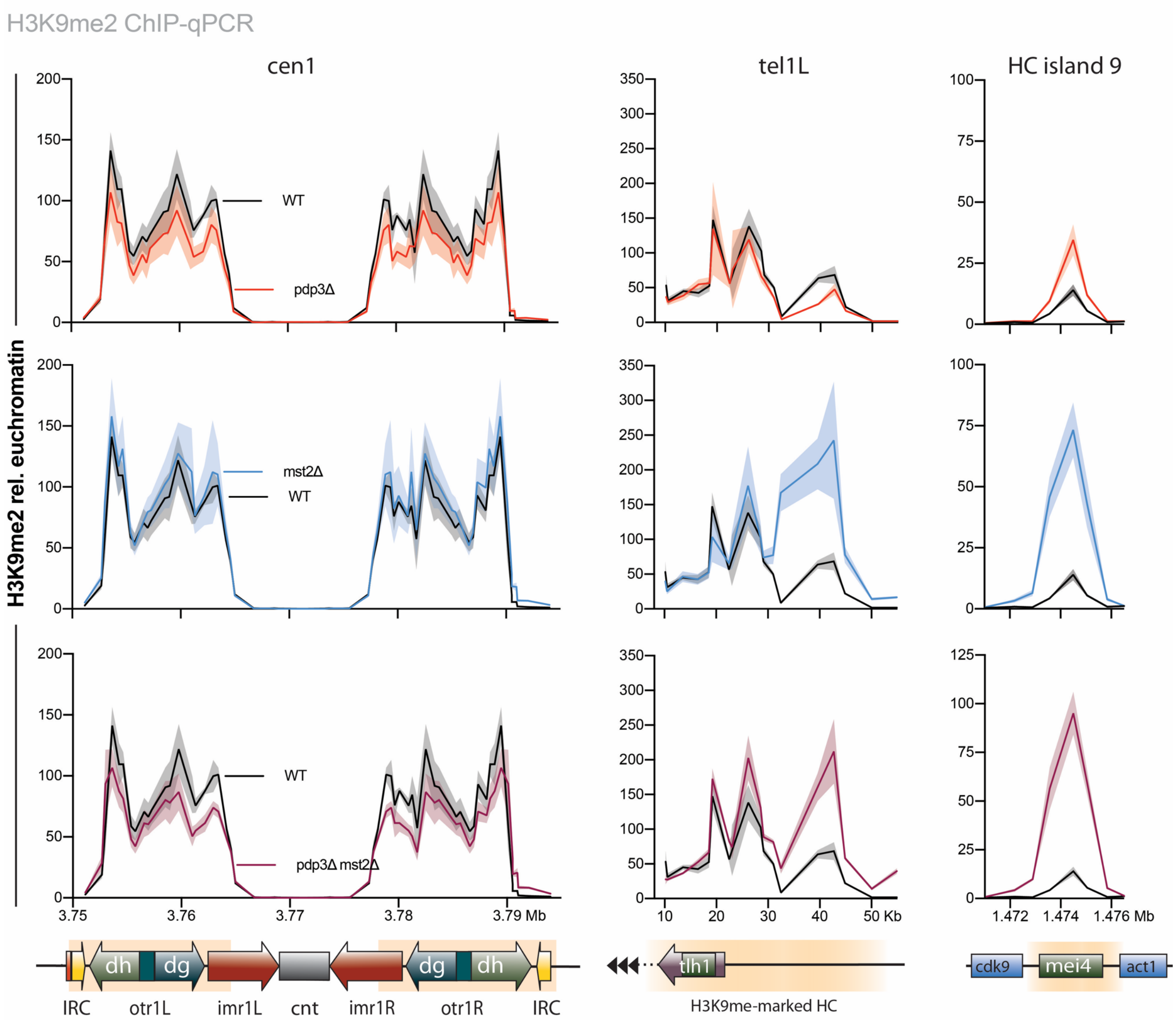

Maintenance and assembly of H3K9me2 at constitutive heterochromatin, subtelomeric boundaries and facultative heterochromatin.

ChIP-qPCR experiments depicting H3K9me2 enrichment at pericentromeric heterochromatin of centromere 1, the subtelomeric TEL1L region, and the *mei4*$^{+}$ locus, a meiotic island, in *pdp3*$^{+}$ and *mst2*$^{+}$ single knockouts as well as an *mst2*$^{+}$ *pdp3*$^{+}$ double knockout; all data have been normalized to the average of three euchromatic genes (*adf1*$^{+}$, *sam1*$^{+}$, *tif51*$^{+}$); n = 3 ± SEM.

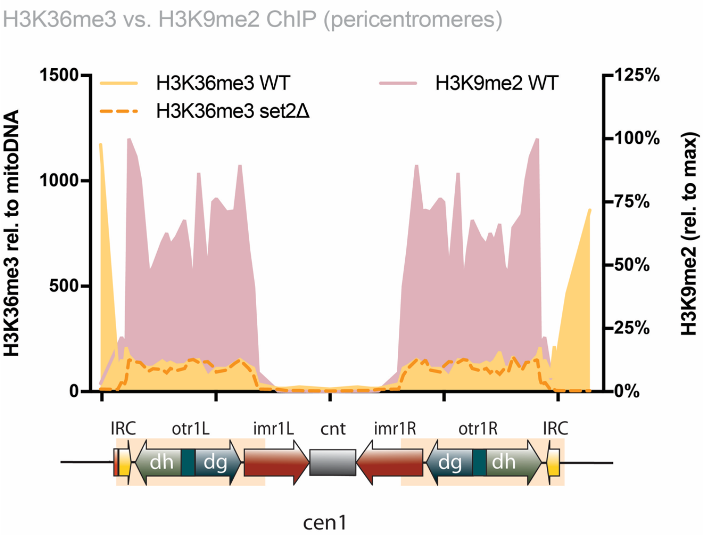

Figure S2: Cross reactivity analysis of anti-H3K36me3 to H3K9me2

Shown is a ChIP-qPCR analysis of a representative experiment using tiling primer arrays for H3K36me enrichments in WT and *set2*Δ cells at the pericentromeric region of chromosome 1. H3K36me3 enrichments were normalized to mitochondrial DNA, which is not affected by histone modifiers (left y-axis). For comparison with H3K9me2, a ChIP-qPCR analysis of WT cells (n = 3) from Barrales et al. 2016 is shown (H3K9me enrichments normalized to maximal level in heterochromatin, which was set to 100%; right y-axis).

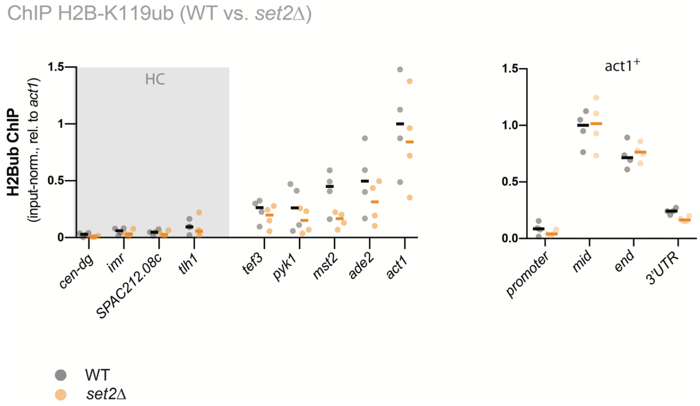

Figure S3: H2BK119 ubiquitylation at heterochromatin and euchromatin

ChIP-qPCR analysis for H2B-K119ub in wild-type and *set2*Δ cells. Circles and horizontal lines represent individual data and median from 4 independent experiments. Input-normalized ChIP data are shown relative to the median of the ChIP signals for *act1*$^{+}$ (mid).

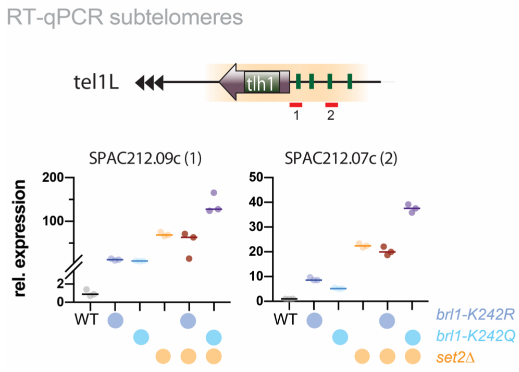

Figure S4: Subtelomeric transcript levels in mutants affect in Brl1 acetylation.

RT-qPCR analysis of transcript levels at subtelomeric HC. Data analysis as in Figure 1C (*n* = 3 individual experiments).

Supplementary Table 1. Yeast strains

STRAIN	SOURCE	IDENTIFIER
h^+ imr1L(NcoI)::$ura4^+$ otr1R(SphI)::$ade6^+$ leu1-32 ura4-DS/E ade6-M210	(Braun et al., 2011)	PSB65
h^+ imr1L(NcoI)::$ura4^+$ otr1R(SphI)::$ade6^+$ leu1-32 ura4-DS/E ade6-M210 set2Δ::natMX	This study	PSB1334
h^- SPSQ (cyhR) SPL42 (cyhS) hphMX::cen1 imr1L(NcoI)::$ura4^+$ otr1R(SphI)::$ade6^+$ leu1-32 ura4-DS/E ade6-M210	(Barrales et al., 2016)	PSB582
h^- SPSQ (cyhR) SPL42 (cyhS) hphMX::cen1 imr1L(NcoI)::$ura4^+$ otr1R(SphI)::$ade6^+$ leu1-32 ura4-DS/E ade6-M210 pdp3Δ::natMX	(Flury et al., 2017)	PSB689
h^- SPSQ (cyhR) SPL42 (cyhS) hphMX::cen1 imr1L(NcoI)::$ura4^+$ otr1R(SphI)::$ade6^+$ leu1-32 ura4-DS/E ade6-M210 mst2Δ::natMX	(Flury et al., 2017)	PSB1122
h^- SPSQ (cyhR) SPL42 (cyhS) hphMX::cen1 imr1L(NcoI)::$ura4^+$ otr1R(SphI)::$ade6^+$ leu1-32 ura4-DS/E ade6-M210 eaf6Δ::natMX	This study	PSB1124
h^- SPSQ (cyhR) SPL42 (cyhS) hphMX::cen1 imr1L(NcoI)::$ura4^+$ otr1R(SphI)::$ade6^+$ leu1-32 ura4-DS/E ade6-M210 nto1Δ::natMX	This study	PSB1127
h^- SPSQ (cyhR) SPL42 (cyhS) hphMX::cen1 imr1L(NcoI)::$ura4^+$ otr1R(SphI)::$ade6^+$ leu1-32 ura4-DS/E ade6-M210 ptf1Δ::natMX	This study	PSB1128
h^- SPSQ (cyhR) SPL42 (cyhS) hphMX::cen1 imr1L(NcoI)::$ura4^+$ otr1R(SphI)::$ade6^+$ leu1-32 ura4-DS/E ade6-M210 ptf2Δ::natMX	This study	PSB1130
h^- SPSQ (cyhR) SPL42 (cyhS) hphMX::cen1 imr1L(NcoI)::$ura4^+$ otr1R(SphI)::$ade6^+$ leu1-32 ura4-DS/E ade6-M210 set2Δ::kanMX	This study	PSB2111
h^- SPSQ (cyhR) SPL42 (cyhS) hphMX::cen1 imr1L(NcoI)::$ura4^+$ otr1R(SphI)::$ade6^+$ leu1-32 ura4-DS/E ade6-M210 pdp3Δ::natMX set2Δ::kanMX	This study	PSB2113
h^- SPSQ (cyhR) SPL42 (cyhS) hphMX::cen1 imr1L(NcoI)::$ura4^+$ otr1R(SphI)::$ade6^+$ leu1-32 ura4-DS/E ade6-M210 nto1Δ::natMX set2Δ::kanMX	This study	PSB2115
h^- SPSQ (cyhR) SPL42 (cyhS) hphMX::cen1 imr1L(NcoI)::$ura4^+$ otr1R(SphI)::$ade6^+$ leu1-32 ura4-DS/E ade6-M210 mst2Δ::natMX set2Δ::kanMX	This study	PSB2131
h^- SPSQ (cyhR) SPL42 (cyhS) hphMX::cen1 imr1L(NcoI)::$ura4^+$ otr1R(SphI)::$ade6^+$ leu1-32 ura4-DS/E ade6-M210 eaf6Δ::natMX set2Δ::kanMX	This study	PSB2321
h^- SPSQ (cyhR) SPL42 (cyhS) hphMX::cen1 imr1L(NcoI)::$ura4^+$ otr1R(SphI)::$ade6^+$ leu1-32 ura4-DS/E ade6-M210 ptf1Δ::natMX set2Δ::kanMX	This study	PSB2323
h^- SPSQ (cyhR) SPL42 (cyhS) hphMX::cen1 imr1L(NcoI)::$ura4^+$ otr1R(SphI)::$ade6^+$ leu1-32 ura4-DS/E ade6-M210 ptf2Δ::natMX set2Δ::kanMX	This study	PSB2325
h leu1-32 ura4-D18 ade6-704 trip1+::ade6+ shade6-250/natMX	(Flury et al., 2017)	PSB2356 (spb426)

h^{-} *leu1-32 ura4-D18 ade6-704 trp1*$^{+}$*::ade6*$^{+}$ *nmt1*$^{+}$*::ade6-hp*$^{+}$*::natMX brl1-K242R*	(Flury et al., 2017)	PSB2357 (spb2982)
h-, leu1-32, ura4-D18, ade6-704, trp1^{+}*::ade6*$^{+}$*, nmt1*$^{+}$*::ade6-hp*$^{+}$*::natMX, brl1-K242Q*	(Flury et al., 2017)	PSB2358 (spb2983)
h leu1-32 ura4-D18 ade6-704 trip1+::ade6+ shade6-250/natMX set2Δ::kanMX	This study	PSB2361
h^{-} *leu1-32 ura4-D18 ade6-704 trp1*$^{+}$*::ade6*$^{+}$ *nmt1*$^{+}$*::ade6-hp*$^{+}$*::natMX brl1-K242R set2Δ::kanMX*	This study	PSB2363
h^{-}*, leu1-32, ura4-D18, ade6-704, trp1*$^{+}$*::ade6*$^{+}$*, nmt1*$^{+}$*::ade6-hp*$^{+}$*::natMX, brl1-K242Q, set2Δ::kanMX*	This study	PSB2365

Supplementary Table 2. Oligonucleotides for Figures 1-4

**TARGET**	**SEQUENCE**	**IDENTIFIER**
cen-dg forward	TGCTCTGACTTGGCTTGTCTT	Sg1020
cen-dg reverse	CCCTAACTTGGAAAGGCACA	Sg1021
cen-dh forward	TGAATCGTGTCACTCAACCC	Sg1022
cen-dh reverse	CGAAACTTTCAGATCTCGCC	Sg1023
act1+ (IV) forward	GATTCTCATGGAGCGTGGTT	Sg1028
act1+ (IV) reverse	CGCTCGTTTCCGATAGTGAT	Sg1029
act1+ (V) forward	AACCCTCAGCTTTGGGTCTT	Sg1030
act1+ (V) reverse	TTTGCATACGATCGGCAATA	Sg1031
ura4+ forward	CAGCAATATCGTACTCCTGAA	Sg1026
ura4+ reverse	ATGCTGAGAAAGTCTTTGCTG	Sg1027
mst2+ forward	GCTTGGATCACCTGAAAAGC	Sg1730
mst2+ reverse	CATCGTCGCATACCATAGATGT	Sg1731
ade2+ forward	AGGCATCTGATCCCAATGAG	Sg2670
ade+ reverse	ATTTTGGATGCCTTGGATGA	Sg2671
tef3+ forward	TGGCCTTCTTAGCCTTTTCA	Sg2736
tef3+ reverse	CTGAGGAAGTTTGGGCTGTC	Sg2737
eno102+ (I) forward	AGCAGGTTGGGGATTGATGG	Sg2759
eno102+ (I) reverse	CAGCTTCTAGCCCAACAGCT	Sg2760
gal1+ (I) forward	TTATGGAGCCAGAACGACGG	Sg2765
gal1+ (I) reverse	GATGTACCGCAGATCCACCC	Sg2766
gal7+ (I) forward	CCACCGTTGTTGAGATCAGC	Sg2772
gal7+ (I) reverse	AGCTGCTTGTTCACTGGTCA	Sg2773
gal10+ (I) forward	GAGTTTCAAAGTCGCAGCGG	Sg2778
gal10+ (I) reverse	GGGAGAATTTCGACCTCGCA	Sg2779
SPAC977.15 (I) forward	TGTGCGCATCCATCTCTTCT	Sg2790
SPAC977.15 (I) reverse	CCAAGCATCAACTTCCTCAGG	Sg2791

tlh1+/tlh2+ (VI) forward	TGCCCCGTACGCTTATCTAC	Sg2940
tlh1+/tlh2+ (VI) reverse	TTGCCTTTCTAGCCCATGAC	Sg2941
SPAC212.09c forward	TCCTTCAGAAATGGCTTGCT	Sg2942
SPAC212.09c reverse	GCATGTGTGTTATCCCGTTG	Sg2943
SPAC212.08c forward	TAATGAGTTGCCCCGGGTAT	Sg2944
SPAC212.08c reverse	CCGAATGGCAAGATGGTAAT	Sg2945
pyk1+ forward	ACGGAAGATGGAGGTGTGAC	N/A
pyk1+ reverse	CCCGTCATCTACGAGAAGGA	N/A

Supplementary Table 3: Oligonucleotides for Figure S1

**TARGET**	**SEQUENCE**	**IDENTIFIER**
mitochondrial DNA forward	ACCAGTACACGAACACGCATT	Sg1742
mitochondrial DNA reverse	ATCCTTCAATCTCCCTCTCCA	Sg1743
IRC-L/R_alt1 forward	TGTCAAGGGAAAAACCGAGA	HC plate
IRC-L/R_alt2 forward	CCCTTGAAGTTTGCCAAAAA	HC plate
ICR-L/R_alt3 forward	CCCGCAAAACCATAAAATGT	HC plate
IRC-L4 forward	TCGTTAGCATTTGGCTTTGA	HC plate
IRC-L2 forward	AACCCAAGCAGATAGACTGAAA	HC plate
cen01 forward	GCAAAGATCGAACGAGTTGTC	HC plate
cen06 forward	TTACCAAATTTGTCAAACGTTAAAT	HC plate
cen07 forward	TGAGGTTTTTCGTTCTTAGGG	HC plate
cen08 forward	TGGACACCACTCTTGCCATA	HC plate
cen10 forward	GGCATTTTGTAAGCGGAAAT	HC plate
cen12 forward	CAGCTTCTTGTACTCACTCACTCA	HC plate
cen16 forward	ATCACGCTTCCTTAGCATGG	HC plate
cen17 forward	ACATTGCTCCGGTGATTTTC	HC plate
cen18 forward	AACCACCACCATGCTCTTTT	HC plate
cen19 forward	TGCGGTCATTTAAAGGCATA	HC plate
cen20 forward	CCCATGATGTCGTTGGTTAAA	HC plate
cen21 forward	ATTTCGCTTTGGCAAAACAT	HC plate

TARGET	SEQUENCE	IDENTIFIER
mitochondrial DNA forward	ACCAGTACACGAACACGCATT	Sg1742
mitochondrial DNA reverse	ATCCTTCAATCTCCCTCTCCA	Sg1743
cen22 forward	TGGAACCCCTAACTTGGAAA	HC plate
cen24 forward	AGAAAATTTCACAACTCCGTTGAT	HC plate
cen25 forward	ACAACATGCAATACCGATTGT	HC plate
cen26 forward	GCACCGTTTTTCCAAATGTC	HC plate
cen27 forward	TCGGAAAATTCATCCTTCAAA	HC plate
cen28 forward	TGAGGTTCATGATGGGTTCA	HC plate
cen29 forward	CGAAGTATGACCCGAATTGC	HC plate
cen30 forward	CGAAAATTGTGTTGTGCCAGT	HC plate
cen31 forward	ATGCTCCGTTGCTTATCTCG	HC plate
cen33 forward	TTTGCATTCTTATCACTTGGATG	HC plate
cen34 forward	GTTTGTTTTGGGGAGACGAA	HC plate
cen35 forward	CCTACCGAACGTATGATTAGCA	HC plate
cen36 forward	CGATCGATTTCTCTTGGTTTTC	HC plate
cen37 forward	CCAAAGCAAATAGTCTAATGATCAAA	HC plate
cen38 forward	CCACCAGACCATTACAAGCA	HC plate
cen39 forward	CGTTGAATGTTGTTGCTTTCA	HC plate
cen40 forward	CATCTCGACTCGCTTGATGA	HC plate
cen41 forward	GTCCTGAATCTTGGCAAACAG	HC plate
cen42 forward	GAAATGGGCAACAAGTCGAT	HC plate
cen43 forward	TCCACTTGGATGACAGAATCC	HC plate
IRC-L/R_alt1 forward	TTGTCACGGTTTGGTTTTCA	HC plate
IRC-L/R_alt2 forward	TTTTCCCTTGACAAAGCTGA	HC plate
ICR-L/R_alt3 forward	TTGGCAAACTTCAAGGGAGT	HC plate
IRC-L/R1 forward	TGCTGAATGTAACCAACATCA	HC plate
IRC-R2 forward	GCAGTGTTTACCAACAAGCGTA	HC plate
CEN1 RB1 forward	ATGCGTTTGCGATTCTCTGC	Sg1787

TARGET	SEQUENCE	IDENTIFIER
mitochondrial DNA forward	ACCAGTACACGAACACGCATT	Sg1742
mitochondrial DNA reverse	ATCCTTCAATCTCCCTCTCCA	Sg1743
		(mb4719)
IRC-R3	TGTGTGTCAAGCAAGAAAGC	HC plate
CEN1 RB2 forward	ACACTGCTTATTCTGCACATGA	Sg1789<br>(mb4721)
CEN1 RB3 forward	AGCCAAACTACATATATTCTCTTCATCG	Sg1791<br>(mb4509)
CEN1 RB4 forward	ACGTACATCTTCGACTAGTTTATCCA	Sg1793<br>(mb4539)
IRC-L/R_alt1 reverse	TGAAAACCAAACCGTGACAA	HC plate
IRC-L/R_alt2 reverse	TTTTCCCTTGACAAAGCTGA	HC plate
ICR-L/R_alt3 reverse	TTGGCAAACTTCAAGGGAGT	HC plate
IRC-L4 reverse	TGCCATATCGTCTTCCGTCT	HC plate
IRC-L2 reverse	TAGGACCGAACTGCCAAAAC	HC plate
cen01 reverse	TGAAATTCCATAAACGGGCTA	HC plate
cen06 reverse	TGCGTTTTCTTAGTAAAAACCTGAT	HC plate
cen07 reverse	GGCAATGTCACAAAGTTTCAA	HC plate
cen08 reverse	TTGCGCATCAAGTATTTTGC	HC plate
cen10 reverse	TGCTTGTTTAGTGTTTGAACGAA	HC plate
cen12 reverse	TCGTTCTTGCCTAGCGAAAT	HC plate
cen16 reverse	TCATTCGTTGTACCAACTGCT	HC plate
cen17 reverse	GGCGTGAATATTGATGTTTTGA	HC plate
cen18 reverse	TCGCAACGATTTGAACTGTC	HC plate
cen19 reverse	CTGTTGTTGAGTGCTGTGGA	HC plate
cen20 reverse	CATGGAGAGCGTATGTTGAAA	HC plate
cen21 reverse	GTTTCCCGCCCAGTAGATG	HC plate
cen22 reverse	TGCTCTGACTTGGCTTGTCTT	HC plate
cen24 reverse	AGAGTTGCCGCAATTGAAAC	HC plate

TARGET	SEQUENCE	IDENTIFIER
mitochondrial DNA forward	ACCAGTACACGAACACGCATT	Sg1742
mitochondrial DNA reverse	ATCCTTCAATCTCCCTCTCCA	Sg1743
cen25 reverse	TCGTTATTGAAACACGAATAGGA	HC plate
cen26 reverse	AACCATTCGCATCCATTTTT	HC plate
cen27 reverse	TCAGCAATTGTTTCAGAAAATG	HC plate
cen28 reverse	TTCGGTCTTTGCAGGACTCT	HC plate
cen29 reverse	CCACGGAAAACAAATTACCG	HC plate
cen30 reverse	CATTCATCTTGCGTGTCTGC	HC plate
cen31 reverse	TCCTCACATTCGACATGACTG	HC plate
cen33 reverse	TGTCTACGTACGCCAGTTGC	HC plate
cen34 reverse	CGATCAAATCGGTCAGTACG	HC plate
cen35 reverse	TGGGATCGCAATTTTTGATT	HC plate
cen36 reverse	TCGCGAACATCAGCATTACT	HC plate
cen37 reverse	CACGGCGATAAGAAATGGA	HC plate
cen38 reverse	CTCGCCTATTTACCGATCCA	HC plate
cen39 reverse	AATGACAAAGGTGCCGAATC	HC plate
cen40 reverse	TGGGCATTCACGAAACATAG	HC plate
cen41 reverse	TACAAGGACTAAGCCCAAGCA	HC plate
cen42 reverse	GTTGCGCAAACGAAGTTATG	HC plate
cen43 reverse	CAACGCATCTACCTCAGCAG	HC plate
IRC-L/R_alt1 reverse	TGTCAAGGGAAAAACCGAGA	HC plate
IRC-L/R_alt2 reverse	CCCTTGAAGTTTGCCAAAAA	HC plate
ICR-L/R_alt3 reverse	CCCGCAAAACCATAAAATGT	HC plate
IRC-L/R1 reverse	GCCTCAATTGCCTATTAGTGCT	HC plate
IRC-R2 reverse	AGAGAATCGCAAACGCATCT	HC plate
CEN1 RB1 reverse	GTGTGAGCGCTAACTTTTGCT	Sg1788 (mb4720)
IRC-R3 reverse	TTCATGTGCAGAATAAGCAGTG	HC plate

TARGET	SEQUENCE	IDENTIFIER
mitochondrial DNA forward	ACCAGTACACGAACACGCATT	Sg1742
mitochondrial DNA reverse	ATCCTTCAATCTCCCTCTCCA	Sg1743
CEN1 RB2 reverse	TGCCGCATGTGGTAAAGACA	Sg1790 (mb4722)
CEN1 RB3 reverse	TTGGCAGAATGTCTAGGTGTAAACTGTG	Sg1792 (mb4510)
CEN1 RB4 reverse	CTATACTGGCTAACCAACTGATGACATTG	Sg1794 (mb4540)

Supplementary Table 4. Antibodies

ANTIBODY	SOURCE	IDENTIFIER
anti-H3K9me2	Abcam	Cat# ab1220
anti-H3K36me3	Abcam	Cat# ab9050
anti-H3 (ChIP)	Active Motif	Cat# 61475
anti-H2BK120ub1	Active Motif	Cat# 39623

Supplementary Table 5. Chemicals, reagents and commercial assays

CHEMICAL OR REAGENT	SOURCE	IDENTIFIER
5-Fluoroorotic Acid (5-FOA)	Thermo Fisher	Cat# 207291-8-4
AEBSF (Pefabloc SC) *	Roche	Cat# 11585916001
ChIP DNA Clean & Concentrator™	Zymo Research	Cat# D5201
cOmplete™ Protease Inhibitor Cocktail	Roche	Cat# 11836145001
Dynabeads Protein G*	ThermoFisher/ Life Technologies	Cat# 10009D
Formaldehyde	Carl Roth	Cat# 4979
G418 sulfate (Geneticin)	Invitrogen/Life Technologies	Cat# 10131027
Hygromycin	Invitrogen/Life Technologies	Cat# 10687010
Leupeptin hemisulfate*	Carl Roth	Cat# CN33
nourseothricin dihydrogen sulfate (NAT)	WERNER BioAgents GmbH	Cat# 5.0
primaQUANT CYBR Master Mix	Steinbrenner Laborsysteme	Cat# SL-9902B

Proteinase K	Roche	Cat# 3115879001
SuperScript III*	ThermoFisher/ Life Technologies	Cat# 18080085
Turbo DNA free	ThermoFisher/ Life Technologies	Cat# AM1907
